# Trends in the Use of Glyphosate Herbicide and Its Relevant Regulations in Taiwan: A Water Contaminant of Increasing Concern

**DOI:** 10.3390/toxics7010004

**Published:** 2019-01-22

**Authors:** Wen-Tien Tsai

**Affiliations:** Graduate Institute of Bioresources, National Pingtung University of Science and Technology, Pingtung 912, Taiwan; wttsai@mail.npust.edu.tw; Tel.: +886-8-7703202; Fax: +886-8-7740134

**Keywords:** glyphosate, consumption market analysis, residual limit, drinking water, regulation, Taiwan

## Abstract

In Taiwan and other countries, glyphosate has been used widely as a non-selective herbicide over 40 years in crop lands and non-crop lands. However, public concerns about its environmental and health risks have increased rapidly because the International Agency for Research on Cancer (IARC) reclassified it as Group 2A (probably carcinogenic to humans) in 2015. From the viewpoints of environmental quality, food security and human health, it is necessary to regulate the release of glyphosate into the environment due to its massive use. The purpose of this case study was to analyze the historical consumption of glyphosate in Taiwan and also summarize its current regulatory measures through multi-ministerial levels. It showed that the sales quantities of glyphosate in Taiwan can be grouped into three stages, which include a ramping period (1984–1992), a stable period (1992–2007), and a declining period (2007–2016). These variations can be correlated with the annual price, manufacturers’ promotion and other non-selective herbicide competitors (i.e., paraquat and glufosinate), as well as the excellent action features of glyphosate. It should be noted that its sales quantities significantly increased from 3200 metric tons in 2015 to 4535 metric tons in 2016 mainly due to the official announcement of paraquat ban effective in February 2019. The core regulations for protecting food security and water quality from the use of glyphosate are based on its residual limits and standards under the authorization of the Food Sanitation Management Act (FSMA) and the Water Pollution Control Act (WPCA), respectively. More importantly, there are occasional reports of contamination by herbicides (including glyphosate) in drinking water sources. Unfortunately, glyphosate is not yet considered among chemical items when evaluating drinking water quality standards in Taiwan.

## 1. Introduction

Herbicides are generally categorized into two groups: selective herbicides and non-selective herbicides [1]. The former ones control specific weed species, while leaving desired crops with limited injury or damage. By contrast, the latter ones totally kill all plants, including undesired weeds and desired crops. Herbicides can also be divided into organic and inorganic chemicals (e.g., sodium chlorate). Based on their chemical structures, organic herbicides include benzene and phenoxy compounds (e.g., 2,4-D), aniline and anilide compounds (e.g., butachlor), urea compounds (e.g., diuron), carbamate compounds (e.g., benthiocarb), diphenylether compounds (e.g., oxyfluorfen), nitrile compounds (e.g., dichlobenil), pyridine compounds (e.g., paraquat), triazine compounds (e.g., atrazine, simazine), dinitroaniline compounds (e.g., pendimethalin), organophosphorous compounds (e.g., glyphosate, glufosinate), and miscellaneous compounds [2]. According to the mechanism of inhibition action (e.g., photosynthesis, pigment synthesis, cell division, and amino acid synthesis), they can be also classified into contact herbicides and translocated herbicides. In Taiwan, the commonly used herbicides include glyphosate, paraquat, butachlor, simazine, diuron, glufosinate, pendimethalin, 2,4-D, and benthiocarb [3]. Like other countries, glyphosate is the most widespread herbicide in Taiwan [4].

Glyphosate, *N*-(phosphonomethyl)glycine, may be the most used herbicide in the world [5]. It is a non-selective, post-emergence and systemic herbicide that controls or kills more weed species than any other herbicide. Its mechanism of action is through the shikimate metabolic pathway, leading to the disruption of aromatic amino acid synthesis [6]. It was introduced in the early 1970s because of its relatively low toxicity, long-lasting efficacy, and weak bioaccumulation. However, this herbicide was not widely used in the commercial market due to its relatively expensive price. As its price significantly declined in the 1980s, it was increasingly adopted in many other fields. In addition, glyphosate-resistant (GR) crops were commercially sold in the mid-1990s, mitigating concerns of crop injury [7]. Combining its low cost with the effectiveness of GR crops led to the wide use of glyphosate in a variety of crops production and plant control (including weeds, grasses, and woody plants) in the past two decades. However, the introduction of GR crops had an adverse effect on the appearance of glyphosate-resistant weeds [8], thus causing agronomic crops to become increasingly vulnerable to weed-resistant infestations. More importantly, its adverse effects on the ecological environment and human health have attracted more and more attention because of its widespread diffusion into the environment and the food chain in the past decade [9].

**Figure 1:**
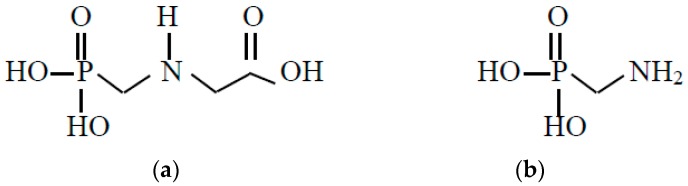
The chemical structures of (**a**) glyphosate and (**b**) AMPA.

Glyphosate is an odorless and white crystalline solid comprised of one basic amino function and three ionizable acidic sites. Due to it limited solubility in water, glyphosate was commercially converted to a more soluble monobasic salt (i.e., isopropylamine, sodium, potassium, trimethylsulfonium, or ammonium) in the aqueous solution at approximately 30–50 wt% [7]. In Taiwan, glyphosate–isopropylamine with 41% (*w*/*w*) was sold on the market in the early 1980s [3]. The physicochemical properties of glyphosate (i.e., low volatility, high density and low octanol–water partition coefficient) indicate that it is not liable to evaporate from the treated surfaces and unlikely to bioaccumulate in higher organisms [10,11]. For instance, it has a low volatility with vapor pressure of about 2.0 × 10^−5^ mmHg at 25 °C. In addition, it is a white, crystalline powder with a specific gravity of 1.7. Although abiotic reaction should not contribute to the degradation of glyphosate in sterile environmental conditions, it degrades readily in soil, non-sterile water and sediments [11]. The main metabolic pathway of glyphosate in soils involves the formations of CO_2_ and aminomethylphosphonic acid (AMPA), which is the main intermediate metabolite [5,12]. Figure 1 shows the chemical structures of glyphosate and AMPA. On the other hand, the fate of glyphosate in soils also includes sorption and leachability [13,14]. Although glyphosate can be dissipated by microbial degradation and adsorbed on humic substances and clay minerals in soils, its leaching seems to be mainly determined by soil structure and rainfall [15]. The transport of glyphosate to the subsurface may be caused by high rainfall events shortly after application on wet sandy soils. It was reported that concentrations of glyphosate in water bodies have been detected in certain rare cases [11]. As a consequence, the subsurface leaching and surface runoff of glyphosate will affect the ground and surface water quality as related to drinking water quality [16]. It is necessary to treat the drinking water sources for degradation and removal of glyphosate and AMPA by effective methods such as advanced oxidation (e.g., ozonation) and activated carbon adsorption [17].

Regarding the toxicity of glyphosate, there was little limited evidence of animal and human health hazards before 2014. However, the International Agency for Research on Cancer (IARC) published a monograph in 2015, concluding that glyphosate is a Group 2A carcinogen based on its limited evidence in humans and sufficient evidence in experimental animals, especially in genotoxicity and oxidative stress [18]. On the other hand, some epidemiologic studies and professional organizations like the European Food Safety Authority (EFSA) declared that it was “unlikely” to be carcinogenic to humans, or that it could cause any type of risk to human health [19,20,21,22,23]. In addition, it was reported that some herbicides, including glyphosate, are potential endocrine disrupting chemicals (EDC) [24,25,26]. In spite of the above-mentioned debate about its carcinogenic classification, the massive use of glyphosate has adverse impacts on the environment that has not only affected the soil, surface and groundwater qualities, but also food safety and the health of farmers [26]. For this reason, some standards for glyphosate residual limits have been established in Taiwan for the purposes of guarding crop foods and livestock and poultry products. More significantly, the risks posed by glyphosates have become of increasing concern around the world with respect to the implementation of a monitoring system [27]. From the viewpoints of environmental fate, this substance may be considered as an emerging contaminant because it could be discharged into the environment by means of wet/dry deposition, leaching, runoff, or infiltration [28], thus causing the deterioration of water quality in the surface water, groundwater, and soils [29]. Additionally, these water bodies can in turn contaminate food crops like fruits and vegetables [30,31,32].

In order to reduce the impacts of glyphosate use on the environmental quality and food chain, this paper submits a Taiwan’s case study on the analysis of glyphosate consumption in the past three decades (1984–2016) and its regulatory countermeasures in foods and water bodies. Based on the above-mentioned aims, the methods in this work were adopted from the data (statistics and regulations) on the use of glyphosate in official and professional websites. They include the central competent authorities (i.e., the Council of Agriculture and the Ministry of Health and Welfare, and the Environmental Protection Administration in Taiwan), and the Weed Science Society (Taiwan).

## 2. Use and Consumption of Glyphosate in Taiwan

Taiwan is a subtropical and tropical country with humid and warm climate. In addition, its population is as high as 2.36 million people, which is equivalent to a population density of about 660 people per km^2^. Therefore, the features of agricultural production are small-scale, continuous, labor-intensive cultivation practice, resulting in the massive use of agrochemicals (e.g., pesticides, herbicides) at higher rates applied to a total cropland of about 750,000 hectares. In Taiwan, there are three main non-selective herbicides accounting for about 50% of the total sales of herbicides in Taiwan’s market [3], which include glyphosate–isopropylamine (41% SL), paraquat (24% SL) and glufosinate–ammonium (13.5/18.5% SL). Herein, the herbicide glyphosate was formulated as “Soluble Liquid” (SL) with 41% (*w*/*w*). Regarding the modes of action and primary uses, photosystem I inhibitors (also referred to as cell membrane disruptors), including paraquat and glufosinate, are commonly used for non-selective weed control and crop desiccation prior to harvest. By contrast, the mode of action for glyphosate is based on the disruption of aromatic amino acid synthesis through the shikimate metabolic pathway.

**Figure 2:**
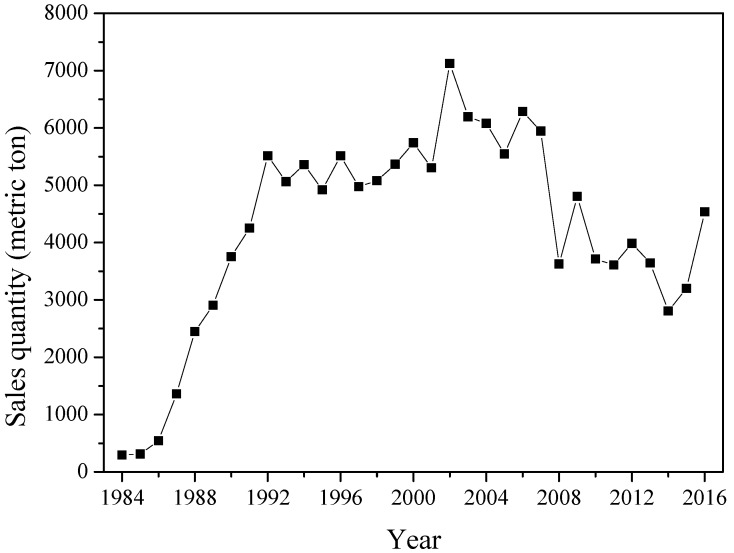
Sales quantities of glyphosate (with isopropylamine salt, 41% solution) in Taiwan since 1984.

Regarding the consumption (sales) quantities of glyphosate in Taiwan, the data were mainly compiled from the Council of Agriculture and the Weed Science Society of the Republic of China. Figure 2 depicts the statistical data on sales quantities of glyphosate during the years of 1984–2016 [3]. Basically, the data can be grouped into three stages.

• Stage I (1984–1992): Ramping period

The sales quantities of glyphosate significantly increased from 294 metric tons in 1984 to 5516 metric tons in 1992. This ramping trend was triggered by the manufacturers lowering the price compared to that of other herbicides (i.e., paraquat).

• Stage II (1992–2007): Stable period

During this period, the price of glyphosate continued to decline slightly, indicating a close price with paraquat [3]. In addition, glyphosate was consistently used in the market because of its special features (i.e., relatively low toxicity, long-lasting efficacy, not liable to bioaccumulation).

• Stage III (2007–2016): Declining period

As shown in Figure 2, the sales quantities of glyphosate indicated a declining trend during this period of 2007–2015. This situation should be attributed to the marketing of another herbicide, glufosinate, in Taiwan during this period. Obviously, both glyphosate and glufosinate–ammonium have dominated the non-selective herbicide market in terms of sales quantities in the past decade. For instance, the sales quantities of glyphosate (41 wt%), glufosinate (18.02 and 13.5 wt%) and paraquat (21 wt%) in 2016 were 4535, 3634 and 2460 metric tons, respectively [3]. Although the price of glufosinate was higher than those of glyphosate and paraquat, glufosinate possesses significant benefits like no weed-resistance and low toxicity. It should be noted that the sales quantities of glyphosate abruptly increased from 3200 metric tons in 2015 to 4535 metric tons in 2016 due to a new regulation preannounced by the central competent authority in 2016. The ban on the use of paraquat has now been formally announced and will become effective from 1 February 2019 to prevent use in self-poisoning.

## 3. Regulatory Management of Glyphosate in Foods and Water Bodies

Regarding the regulatory frameworks for reducing the pesticide risks in Taiwan, this task must be a joint venture by the multi-ministerial levels, which includes the central competent authorities such as the Council of Agriculture (COA), the Environmental Protection Administration (EPA), and the Ministry of Health and Welfare (MOHW). The central authority COA focuses on the management measures of all pesticides, including registration, manufacturing, import, export, sale and use or restriction (ban) under the Pesticide Management Act (PMA) recently revised on 23 May 2018. The Act was enacted to protect agricultural production and the ecological environment, prevent and eliminate pests, prevent hazards from pesticides, strengthen pesticide administration, promote the development of the pesticide industry, and enhance the safety of agricultural products like crops and foods. Although glyphosate was listed as a Group 2A carcinogen by the IARC in 2015 and some court cases have begun due to its use [18], the COA has no plans to ban the use of glyphosate. However, the COA and EPA have been working together to reduce the use of pesticides in non-agricultural areas, including schools, parks and graveyards. In 2017, the COA announced a 10-year plan to cut domestic pesticide use in half by 2027. In this regard, the Organic Agriculture Promotion Act was thus enacted on 30 May 2018, aiming at maintaining water and soil resources, the ecological environment, biodiversity, animal welfare and consumer interests, and promoting agricultural operation that is eco-friendly and sustainable use of resources. The COA promotes the agricultural production management system which adopts the approaches of agronomical, biological and machinery operation, and also uses natural resources, excluding synthetic chemicals (e.g., pesticides, chemical fertilizers) and genetically modified organism (GMO) crops, in order to fulfill the eco-friendly requirement of organic agriculture.

Because of its widespread diffusion into the environment, glyphosate will be found in water bodies, soils or sediments which could be incorporated to the water and food supply chains [28]. Therefore, herbicides like glyphosate may enter into human tissues through contaminated foods, thus increasing potential health risks [26]. In order to protect public health, the maximum residue limit (MRL) for pesticides have been promulgated around the world. For instance, the Ministry of Health, Labour and Welfare (MHLW) in Japan has set the maximum residue limits for glyphosate in a variety of agricultural products [33]. In Taiwan, the central authority MOHW has established many regulations to ensure the sanitation, safety and quality standards for food and its storage and package containers under the authorization of the Food Sanitation Management Act (FSMA). Currently, there are two relevant regulations (i.e., “Residual Limits of Pesticides in Livestock and Poultry Products” and “Residual Limits of Pesticides in Foods”) pertaining to the regulatory management of pesticides in the livestock and poultry products (including meat, milk and eggs) and crops, respectively. Table 1 lists the glyphosate residue limits in crops and livestock and poultry products under the authorizations of the allied regulations in Taiwan. By comparing MRL values between Taiwan and Japan, it was found that the MRLs for glyphosate in Taiwan are larger than those in Japan, but more stringently regulated in Taiwan.

On the other hand, this regulation has been developed to protect human health due to the release of glyphosate into the water and soil environments based on the regulatory frameworks by the EPA, because it is the most widely used herbicide in Taiwan. Table 2 summarizes the environmental standards/limits of glyphosate under the authorization of the Water Pollution Control Act (WPCA). In recent years, the EPA was considering adding glyphosate as a chemical item under the authorizations of the Soil and Groundwater Pollution Remediation Act (SGPRA) and the Drinking Water Management Act (DWMA) because concentrations of glyphosate in water bodies and soils have been detected in some countries or regions [34,35,36,37]. For instance, it was detected in runoff from a highway (California, USA) in concentrations ranging from 1.36 to 9.44 μg/L [11]. Actually, human exposure to glyphosate by contact with soil and plants may not occur because it will be rapidly inactivated by its strong adsorption into soil particles. The epidemiological studies of glyphosate show no consistent patterns associated with cancer and non-cancer risks [19,38]. Regarding the drinking water quality standards, it will be discussed in the subsequent section.

## 4. Drinking Water Quality Standards in Taiwan

In Taiwan, the Drinking Water Quality Standards (DWQS) were promulgated under the authorization of the DWMA, which aims at ensuring the quality of drinking water sources, improving public drinking water quality and maintaining public health. On 10 January 2017, the EPA announced the revision of the DWQS. The updated standards were categorized into biological, physical and chemical groups. The chemical standards in the DWQS include the following substances:

- Substances that impact health (44 items).
  Including arsenic, lead, selenium, total chromium, cadmium, barium, antimony, nickel, mercury, cyanide, nitrite-nitrogen, disinfection byproducts (total trihalomethanes, haloacetic acids, bromate, chlorite), volatile organic compounds, agricultural chemicals (endosulfan, lindane, butachlor, dichlorophenoxyacetic acid, paraquat, methomyl, carbofuran, isoprocarb, methamidophos, diazinon, parathion, EPN, monocrotophos), and persistent organic compounds (dioxins).
- Substances with potential health impact (5 items).
  Including fluoride, nitrate-nitrogen, silver, molybdenum, and indium.
- Contaminants that cause aesthetic, cosmetic, and technical effects (12 items).
  Including iron, manganese, copper, zinc, sulfate, phenols, anionic surfactants, chloride, ammonia–nitrogen, total hardness as CaCO_3_, total dissolved solids, and aluminum.
- Residual chlorine
- pH

In fact, the DWQS were originally promulgated in 1998 and have been amended six times since then. At present, there are 68 chemical items controlled under the standards. Over the years, the EPA has taken into account environmental changes, technological development and international regulation trends and restrictions adopted by other developed nations when reviewing the standards. The EPA took the following factors into account when drafting the latest amendments:

- The current drinking water quality monitoring situation in Taiwan.
- Drinking water standards adopted by developed nations and organizations, including Australia, Canada, the EU, Japan, New Zealand, the UK, the US, and the WHO.
- Toxicological data and other relevant regulations for emerging contaminants.
- Risk assessment information of each of the drinking water standards used by the above-mentioned countries.

Obviously, the chemical items in the DWQS include some herbicides such as paraquat, butachlor, and dichlorophenoxyacetic acid (2,4-D). Although glyphosate is rapidly inactivated by strong sorption onto clay soil particles, it has been detected in water supply sources like groundwater and surface water [34,35,36,37]. Therefore, some countries established the exposure standards and guidelines of glyphosate. For example, the drinking water standards of glyphosate have a maximum acceptable concentration in Canada and Australia of 0.28 and 1.0 mg/L, respectively. The US drinking water standard of glyphosate maximum contaminant level (MCL) is 0.7 mg/L. Noticeably, the MCL of glyphosate was not yet formulated by nearby countries like Korea and Japan [39,40]. Due to the massive use of glyphosate and its environmental and health risks as mentioned above, it was listed by the EPA in Taiwan as one of the priority candidate chemicals in the DWQS. Furthermore, the maximum allowable concentration (MAC) of glyphosate in drinking water was suggested by the EPA as 0.9 mg/L based on the usage amounts, toxicological data, environmental residual levels, and acceptable daily intake (ADI, 0.3 mg kg^−1^ day^−1^) [11]. Subsequently, this candidate was not blanketed into the standards because it was not found at tap water purification plants [41]. It should be noted that the concentrations of glyphosate and AMPA (the main metabolite of glyphosate) have been identified as high as about 100 μg/L in surface waters [35].

## 5. Conclusions and Recommendations

In recent years, many emerging contaminants (e.g., endocrine disrupting chemicals, personal care products, pharmaceuticals) have been found in environmental media with the advance of technology for chemical analysis. In this regard, glyphosate is widely used as a non-selective herbicide in a variety of fields, including crop lands, public areas, homes and gardens. Humans could be exposed to it and its metabolites through dietary foods and media (water, soil, air). More significantly, the International Agency for Research on Cancer (IARC) reclassified glyphosate as Group 2A (Probably carcinogenic to humans) in 2015. In order to protect human health, the multi-ministry organizations in Taiwan have promulgated relevant regulations for setting the residual limits of glyphosate in livestock and poultry products (including meat, milk and eggs) and crops. Although the COA took measures like biological pest control, organic farming promotion and regulatory control, the sales quantities of glyphosate in Taiwan still ranged from 2800 metric tons to 4800 metric tons in the past decade (2007–2016). In addition, the expectable use of glyphosate in Taiwan will be more extensive when the ban on the use of paraquat becomes effective in February 2019. On the other hand, glyphosate is not yet covered among the chemical items when evaluating drinking water quality standards in Taiwan. Due to its probably carcinogenic toxicity, massive use and residues in drinking water sources, the EPA should take the following actions for protecting human health as follows:

- Adopting advanced instruments for analyzing trace levels (i.e., ppbw levels) of residual glyphosate in common crops and drinking water sources.
- Evaluating health risk of exposure to glyphosate through dietary foods and media (especially water), and re-establishing an acceptable daily intake.
- Adding glyphosate to the chemical items of the DWQS or the water quality standards of drinking water sources.

## Figures and Tables

**Table 1:** Glyphosate residue limits in crops and livestock and poultry products in Taiwan.

Residue Limits				
Crop	Value (ppm)	Residual Tissue	Animal Species	Value (ppmw)
Almonds	1.0	Muscle	Cattle, pig, poultry	0.1
Asparaguses	0.5	Edible offal	Cattle	2
Citrus	0.1	Milk	Cattle	0.1
Corns	1.0	Edible offal	Pig	1
Drupe	0.2	Egg	--	0.1
Other dry beans ^a^	2.0	--	--	--
Large berries	0.2	--	--	--
Lentil (dry)	5.0	--	--	--
Pea (dry)	5.0	--	--	--
Peppers	0.1	--	--	--
Pome	0.2	--	--	--
Potatoes	0.2	--	--	--
Prunes	0.1	--	--	--
Rice	0.1	--	--	--
Small berries	0.2	--	--	--
Soybeans	10	--	--	--
Sugarcane	0.1	--	--	--
Sunflower seed	7.0	--	--	--
Tea	0.1	--	--	--
Vegetable soybeans	0.2	--	--	--
Wheat	5	--	--	--

^a^ Except lentil, sunflower seed, pea, soybean.

**Table 2:** Environmental standards/maximal limits of herbicides in water bodies in Taiwan.

Water Bodies	Standards/Limits (mg/L)
Discharge water (Effluent) ^a^	1.0
Groundwater (Injection) ^a^	0.1

^a^ Total amount of seven herbicides (butachlor, paraquat, 2,4-D, alachlor, chlornitrofen, glyphosate and diquat).

## References

[1] Carlile B. (2006). Pesticide Selectivity, Health and the Environment.

[2] Ware G.W. (1989). The Pesticide Book.

[3] Fong R.L.P. (2017). Herbicide market in Taiwan for 60 years (in Chinese). Weed Sci. Bull..

[4] Richmond M.E. (2018). Glyphosate: A review of its global use, environmental impacts, and potential health effects on humans and other species. J. Environ. Stud. Sci..

[5] Leyva-Soto L.A., Balderrama-Carmona A.P., Moran-Palacio E.F., Diaz-Tenorio L.M., Gortares-Moroyoqui P. (2018). Glyphosate and aminomethylphosphonic acid in population of agricultural fields: Health risk assessment overview. Appl. Ecol. Environ. Res..

[6] Dill G.M., Sammons R.D., Feng P.C.C., Kohn F., Kretzmer K., Mehrsheikh A., Bleeke M., Honegger J.L., Farmer D., Wright D., Nandula V.K. (2010). Glyphosate: Discovery, development, applications and properties. Glyphosate Resistance in Crops and Weeds: History, Development, and Management.

[7] Green J.M. (2009). Evolution of glyphosate-resistant crop technology. Weed Sci..

[8] Heap I., Duke S.O. (2018). Overview of glyphosate-resistant weeds worldwide. Pest. Manag. Sci..

[9] Torretta V., Katsoyiannis I.A., Viotti P., Rada E.C. (2018). Critical review of the effects of glyphosate exposure to the environment and humans through the food supply chain. Sustainability.

[10] Farmer D., Krieger R. (2010). Inhibitors of aromatic acid biosynthesis. Hayes’ Handbook of Pesticide Toxicology.

[11] Baer K.N., Marcel B.J., Wexler P. (2014). Glyphosate. Encyclopedia of Toxicology.

[12] Silva V., Montanarella L., Jones A., Fernandez-Ugalde O., Mol H.G.J., Ritsema C.J., Geissen V. (2018). Distribution of glyphosate and aminomethylphosphonic acid (AMPA) in agricultural topsoils of the European Union. Sci. Total Environ..

[13] Vereecken H. (2005). Mobility and leaching of glyphosate: A review. Pest. Manag. Sci..

[14] Borggaard O.K., Gimsing A.L. (2008). Fate of glyphosate in soil and the possibility of leaching to ground and surface waters: A review. Pest. Manag. Sci..

[15] Sviridov A.V., Shushkova T.V., Ermakova I.T., Ivanova E.V., Epiktetov D.O., Leontievsky A.A. (2015). Microbial degradation of glyphosate herbicides (Review). Appl. Biochem. Microbiol..

[16] Noori J.S., Dimaki M., Mortensen J., Svendsen W.E. (2018). Detection of glyphosate in drinking water: A fast and direct detection method without sample pretreatment. Sensors.

[17] Josson J., Camm R., Hall T. (2013). Removal and degradation of glyphosate in water treatment: A review. J. Water Supply Res. Technol. AQUA.

[18] International Agency for Research on Cancer (IARC) IARC Monographs Volume 112: Evaluation of Five Organophosphate Insecticides and Herbicides. https://monographs.iarc.fr/wp-content/uploads/2018/06/mono112-10.pdf.

[19] Mink P.J., Mandel J.S., Sceurman B.K., Lundin J.I. (2012). Epidemiologic studies of glyphosate and cancer: A review. Regul. Toxicol. Pharmcol..

[20] Brusick D., Aardema M., Kier L., Kirkland D., Williams G. (2016). Genotoxicity Expert Panel review: Weight of evidence evaluation of the genotoxicity of glyphosate, glyphosate-based formulations, and aminomethylphosphonic acid. Crit. Rev. Toxicol..

[21] Williams G.M., Aardema M., Acquavella J., Berry S.C., Brusick D., Burns M.M., de Camargo J.L.V., Garabrant D., Greim H.A., Kier L.D. (2016). A review of the carcinogenic potential of glyphosate by four independent expert panels and comparison to the IARC assessment. Crit. Rev. Toxicol..

[22] Tarazona J.V., Court-Marques D., Tiramani M., Reich H., Pfeil R., Istace F., Crivellente F. (2017). Glyphosate toxicity and carcinogenicity: A review of scientific basis of the European Union assessment and its differences with IARC. Arch. Toxicol..

[23] Davoren M.J., Schiestl R.H. (2018). Glyphosate-based herbicides and cancer risk: A post-IARC decision review of potential mechanisms, policy and avenues of research. Carcinogenesis.

[24] Gasnier C., Dumont C., Benachour N., Clair E., Chagnon M.C., Séralini G.E. (2009). Glyphosate-based herbicides are toxic and endocrine disruptors in human cell lines. Toxicology.

[25] Mnif W., Hassine A.I.H., Bouaziz A., Bartegi A., Thomas O., Roig B. (2011). Effect of endocrine disruptor pesticides: A review. Int. J. Environ. Res. Public Health.

[26] Van Bruggen A.H.C., He M.M., Shin K., Mai V., Jeong K.C., Finckh M.R., Morris J.G. (2018). Environmental and health effects of the herbicide glyphosate. Sci. Total Environ..

[27] Steimer M., Gerbl-Rieger S., Schaff P., Parlar H. (2007). Current situation of the application and identification of pesticides in EU bordering countries with respect to the implementation of a monitoring system. Fresenius Environ. Bull..

[28] Starrett S.K., Klein J., Nett M.T., Carroll M.J., Horgan B.P., Petrovic A.M. (2008). Glyphosate runoff when applied to Zoysiagrass under golf course fairway conditions. The Fate of Nutrients and Pesticides in the Urban Environment.

[29] Fawell J., Ong C.N., Tortajada C. (2013). Emerging contaminants and the implications for drinking water. Water Quality Policy and Management in Asia.

[30] Cerdeira A.L., Duke S.O. (2006). The current status and environmental impacts of glyphosate-resistant crops: A review. J. Environ. Qual..

[31] Solomon K.R., Marshall E.J.P., Carrasquilla G. (2009). Human health and environmental risks from the use of glyphosate formulations to control the production of coca in Colombia: Overview and conclusions. J. Toxicol. Environ. Health A.

[32] Von Merey G., Manson P.S., Mehrsheikh A., Sutton P., Levine S.L. (2016). Glyphosate and aminomethylphosphonic acid chronic risk assessment for soil biota. Environ. Toxicol. Chem..

[33] The Japan Food Chemical Research Foundation Maximum Residue Limits (MRLs) List of Agricultural Chemicals in Foods. http://db.ffcr.or.jp/front/.

[34] Stewart M., Olsen G., Hickey C.W., Ferreira B., Jelic A., Petrovic M., Barcelo D. (2014). A survey of emerging contaminants in the estuarine receiving environment around Auckland, New Zealand. Sci. Total Environ..

[35] Alonso L.L., Demetrio P.M., Etchegoyen M.A., Marino D.J. (2018). Glyphosate and atrazine in rainfall and soils in agroproductive areas of the pampas region in Argentina. Sci. Total Environ..

[36] Demonte L.D., Michlig N., Gaggiotti M., Adam C.G., Beldomenico H.R., Repetti M.R. (2018). Determination of glyphosate, AMPA and glufosinate in dairy farm water from Argentina using a simplified UHPLC-MS/MS method. Sci. Total Environ..

[37] Masiol M., Gianni B., Prete M. (2018). Herbicides in river water across the northeastern Italy: Occurrence and spatial patterns of glyphosate, aminomethylphosphonic acid, and glufosinate ammonium. Environ. Sci Pollut. Res..

[38] Mink P.J., Mandel J.S., Lundin J.I., Sceurman B.K. (2011). Epidemiologic studies of glyphosate and non-cancer health outcomes: A review. Regul. Toxicol. Pharmcol..

[39] Ministry of Environment (Korea) Drinking Water Management. http://eng.me.go.kr/eng/web/index.do?menuId=299&findDepth=1.

[40] Ministry of Health, Labour and Welfare (Japan) Water Supply in Japan. https://www.mhlw.go.jp/english/policy/health/water_supply/menu.html.

[41] Whang L.M. (2017). Examination and evaluation of drinking water sources and drinking water quality standard items (in Chinese). Proceedings of the Environmental Technology Forum.

